# The dialectic of human-object relations in Chinese urban television drama: a reification-theoretical reading

**DOI:** 10.3389/fsoc.2026.1784028

**Published:** 2026-10-05

**Authors:** Wen Liang

**Affiliations:** Yuncheng University, Yuncheng, China

**Keywords:** Chinese television drama, dereification, Lukács, people-centered narration, reification, subjectivity, urban life

## Abstract

**Background:**

Lukács’s theory of reification describes a social order in which relations among people appear as relations among things. It offers a productive way to read urban television narratives, where housing, consumption, professional performance, and intimate life regularly become measures of social worth.

**Objective:**

This article examines how Chinese television dramas make reification visible through objects, space, and the body. It also asks how people-centered narration is constructed as a textual possibility for questioning those relations.

**Methods:**

The study uses qualitative close reading of three urban dramas - Fake It Till You Make It, Nothing But Thirty, and Women in Beijing - and Meet Yourself, a contrastive case that begins with urban workplace pressure before relocating its protagonist to a rural community. Plot events, dialogue, character action, settings, and recurring motifs are examined through object alienation, reified space, the reified body, and dereifying practice.

**Results:**

Across the urban cases, luxury goods, exclusive residences, professional schedules, and intimate calculations turn social value into visible and comparable signs. The contrastive case frames a partial interruption of that logic through care, shared activity, and a changed relation to work. These are narrative resolutions, not demonstrations of institutional transformation.

**Conclusion:**

The article connects reification theory to the close analysis of Chinese television narrative. It argues that people-centered narration is most useful as an analytical term when it names concrete formal and relational practices rather than a general moral quality.

## 1 Introduction

Chinese television dramas about urban life often organize social aspiration through apartments, workplaces, restaurants, branded goods, and intimate partnerships. Such scenes make it possible to study how economic value enters ordinary judgment: who appears credible, who gains access, and whose time or body is treated as valuable.

Research on Chinese realist television has emphasized emotional presentation and the narrative importance of ordinary experience (Ouyang and Xu, 2019). Scholarship has also considered urban identity, gender, and social change. Less attention has been given to the human-object relation as a narrative problem: the processes through which objects, spaces, and bodies become media for classifying people and organizing social relations.

Reification theory makes that problem analytically precise. Although it has been extended to digital media, the Internet, and technological society (Fuchs, 2015, 2016), its use in close readings of Chinese television drama remains comparatively underdeveloped. This article therefore asks how reification is made narratively visible in a small, purposively selected corpus.

The article combines Lukács’s account of reification with Feenberg’s practice-oriented reading of reification and dereification. It treats television drama neither as a transparent record of social life nor as a self-contained aesthetic object, but as a narrative site where social classifications are staged, experienced, and sometimes contested.

Accordingly, this study addresses the following research questions:

RQ1: How do the selected television dramas represent reification under commodity logic?

RQ2: Through which narrative situations are reifying practices reproduced via objects, space, and the body?

RQ3: How do the narratives construct qualified possibilities for dereifying practice and the reconstruction of subjectivity?

The article’s contribution is twofold. It translates reification into textual categories suitable for close television analysis, and it specifies people-centered narration as an author-defined analytical construction rather than an unexamined evaluative label.

## 2 Theoretical framework

For Lukács, the universalization of the commodity form makes relations among people appear as relations among things. Human activity is organized as a system of calculable and exchangeable units, while people encounter that system as an objective order external to their will. Reification therefore concerns both social relations and the forms of consciousness through which those relations are experienced (Lukács, 1971, pp. 83–93).

Feenberg shifts attention from reification as a purely ideological effect to reification as a problem of social practice and social ontology (Feenberg, 2011, 2020). In this account, reification is produced and reproduced in routine action rather than existing as a static condition.

In the present study, dereification does not mean a final escape from modernity, work, or material life. It names situations in which the rules of efficiency, competition, and calculability are made visible and partially reorganized through concrete ways of living, working, and relating to others. The term is used to identify a narrative possibility, not to claim that a television ending changes the institutions that produce inequality or consumer anxiety.

This article uses people-centered narration as an author-defined analytical term. The broader vocabulary of people-centered literature and art in China emphasizes ordinary life, shared experience, and human development (Xi, 2021; Tan, 2023). Here, however, the term is deliberately narrower: it identifies two observable narrative moves - recovering experience obscured by commodity signs, and rebuilding relationships through care, ethical commitment, and shared activity. It does not designate a general moral quality of a drama.

### 2.1 How reified relations take shape: commodity signs, spatial order, and bodily practice

This article treats object alienation, reified space, and the reified body as traces of the same social relation at different points, not as three separate themes. Reification is not a quality that belongs to an object by itself. It arises when relations among people are mediated by a measure that permits comparison and exchange. In these dramas, the measure may be the price or scarcity of a commodity, the status attached to an address, or the expectation that someone should always be available for work. The analysis therefore follows more than the presence of an object: it asks how an object enters a judgement, where that judgement gains force, and what adjustments it demands of the character.

At the level of objects, a complex relationship is compressed into a sign that can be recognized quickly. A handbag, a protective film on the sole of a shoe, or the details of a dinner cannot account for a person’s whole history. They turn someone into an object of quick classification only when they stand in for a fuller encounter. Space gives such signs public force. A residence, restaurant, or workplace places them within a shared situation, distributes access, and establishes who can read them. Taste that appears personal is thus never produced by individual choice alone.

Space also turns a single judgement into an order that can be enacted repeatedly. To appear at ease within it, a character must learn the relevant way of speaking, scheduling time, and managing appearance; these demands then return in work and intimate relations. The body bears and carries out the rules of space. It is not a third sign standing beside objects and space, but the record of expectations already internalized at those two levels. Fatigue and refusal can, in turn, reveal the limits of the order.

For this reason, a scene counts as evidence of a reifying mechanism only when the narrative connects these levels, or presents their connection as a problem. The results begin with objects because they are easiest to see; they then follow the spatial force those objects acquire and the way that force enters bodily practice. This order serves the argument and does not reproduce the chronology of the plot.

## 3 Methodological framework and analytical approach

### 3.1 Research design and case selection

This study uses qualitative close reading to examine how television narratives organize relations between people and things. Its aim is interpretive rather than statistical: it asks how particular scenes make commodity logic intelligible in ordinary life. The corpus consists of Fake It Till You Make It, Nothing But Thirty, and Women in Beijing, with Meet Yourself as a contrastive case. The latter begins in a high-pressure Beijing workplace before moving its protagonist to a rural community; it makes a shift in the character’s relation to work and everyday time narratively available. The cases were selected because they were publicly released, sustained their engagement with contemporary urban experience, and provided substantial material on consumption, work, emotional interaction, and individual development. The sample supports theoretical comparison; it does not claim to represent Chinese television, urban residents, or viewer responses as a whole.

### 3.2 Materials, units of analysis, and procedure

The analytical materials are the released episodes of the four dramas. The close readings attend to plot events, dialogue, character action and interaction, settings, and recurring motifs concerning consumption, work, spatial practice, bodily management, intimacy, and changes in everyday life. Table 1 specifies the distinct role each case plays in the comparison.

**Table 1:** Corpus and analytic role.

Drama	Original title	Initial release	Analytic role
Fake It Till You Make It	装腔启示录	2023	Urban professional life, cultural distinction, and managed availability
Nothing But Thirty	三十而已	2020	Housing, status consumption, bodily display, and intimate calculation
Women in Beijing	北京女子图鉴	2018	Urban advancement, workplace distinction, and visible consumption
Meet Yourself	去有风的地方	2023	Contrastive case: care, community practice, and reconstructed subjectivity

The analysis is limited to these four primary texts. Meet Yourself is used principally for the discussion of dereifying practice; the three urban dramas provide the main evidence for object alienation, reified space, and the reified body.

The unit of analysis is a narrative unit: a plot event, interaction, line of dialogue, setting, or recurring symbolic element that bears on reification or dereification. Each unit is interpreted in its immediate narrative context and in relation to a character’s development. The study does not treat isolated images as self-sufficient evidence.

The analysis proceeded in two interpretive passes. The first identified narrative units involving commodities, urban spaces, bodily management, workplace routines, and intimate relationships. The second compared those units across the corpus through the three dimensions of object alienation, reified space, and the reified body. A final reading traced occasions on which a character questions or reorganizes rules of status, efficiency, and exchange. This is a qualitative interpretive procedure, not a frequency-based coding study.

### 3.3 Conceptual definitions and evidential standards in scene analysis

Operationalization here does not turn close reading into numerical coding. It sets a threshold for the use of theoretical terms. For each candidate scene, the analysis asks first what object or measurable quality is used to judge a character; it then asks what setting gives that judgement practical consequence, what bodily adjustment it demands, and whether the narrative confirms, complicates, or resists the judgement. Substitution at the level of objects corresponds to object alienation; the distribution of access corresponds to reified space; pressure to manage oneself corresponds to the reified body. Dereification becomes possible only where the rules of judgement themselves are loosened.

These questions also provide grounds for exclusion. A handbag used only to indicate personal taste, an expensive apartment that merely serves as a backdrop, or a busy schedule that supplies plot information does not, by itself, demonstrate reification. A scene enters the evidential field only when a sign changes how a character is addressed or treated, a place grants or denies participation, or a character treats time, appearance, feeling, or intimacy as resources to be optimized. This keeps the critique of reification precise without treating every act of consumption as negative.

A single scene may engage all three processes. The primary category is therefore determined by the relation that changes most directly: a change in recognition belongs to the object dimension; a change in access belongs to space; a change in self-management belongs to the body. The other links remain secondary cues rather than being added as frequencies. For each example, the analysis records textual cues, social effects, character responses, and possible counter-evidence. Its interpretations can thus be traced back to particular scenes, without claiming statistical reliability.

## 4 Results

### 4.1 Object alienation

Object alienation appears when things are valued less for use than for their capacity to display identity, status, and belonging. This distinction between use value and sign value is central to accounts of consumer culture (Baudrillard, 2000; Lury, 2003).

A dinner sequence in Fake It Till You Make It stages an ostensibly intimate gathering through a calligraphy-written menu, detailed discussion of ingredients and tableware, and the selection of a demanding art film. The scene turns taste into a social performance: culinary knowledge and cultural reference function as evidence of refinement rather than as private preference.

Nothing But Thirty provides a related case. In the interaction between Liang Zhengxian and Wang Manni, a minor material detail - the sole protector on her high heels - becomes a shortcut for classifying her as an ‘economy-class passenger.’ The scene reduces a person to a quickly decoded consumer sign, even though her appearance otherwise communicates affluence.

Gu Jia’s entry into the wives’ circle makes the same mechanism more explicit. After moving to Junyue Mansion, she confronts a hierarchy organized around housing, luxury goods, and family business. Her purchase of a limited-edition handbag following exclusion from a group photograph shows how an object can mediate access to an otherwise interpersonal relationship.

These scenes are not a moral indictment of consumption. They show something more specific: before a relationship begins, a person is already read through a few visible things. The protective film, the handbag, and the performance of taste at dinner do not have the same economic value, but within their respective circles they all abbreviate an encounter into an act of recognition and make further knowledge of the person seem unnecessary. Because such recognition must be legible in a particular space, object alienation cannot operate on its own; it requires rules of entry before it can become a durable social difference.

### 4.2 Reified space

In the selected urban dramas, housing and consumption venues do more than provide settings. They mark social position, regulate access to networks, and give characters a visible way to assess one another. Reified space is most apparent when a residence or venue is treated as evidence of a person’s worth.

A luxury apartment, central business district, or exclusive venue does not simply contain social interaction; it helps determine who is recognized, who is excluded, and which forms of conduct appear appropriate. Space thus becomes a material shorthand for relations that are otherwise social and historical.

In Nothing But Thirty, Junyue Mansion functions as more than an address. Once Gu Jia enters this residential environment, the apartment’s name becomes a credential within the wives’ circle. Housing and luxury goods operate together as conditions of visibility and access.

In Fake It Till You Make It, upscale restaurants and private gatherings provide a setting in which wine, clothing, conversation, and cultural knowledge signal professional standing. Women in Beijing presents a related pattern of urban aspiration, in which visible consumption and workplace distinction shape the terms of social advancement. In both dramas, the signs are treated as practical conditions of recognition rather than neutral background detail.

Space thus participates in the classification of social relations, but it does more than divide characters into insiders and outsiders. It makes the meaning of things visible and requires adjustments in speech, time, and appearance if a character is not to seem out of place. Here, the sign carried by an object becomes a shared rule of interaction, and the external boundary of space enters the body’s work of self-adjustment. The following discussion of the reified body follows this movement.

### 4.3 The reified body

The reified body is examined here through workplace time, self-presentation, and intimate evaluation. The question is not whether a body is represented, but whether it is made measurable, manageable, or exchangeable within a relation of status and performance.

In Fake It Till You Make It, professional bodies are organized through availability, client demands, and schedules. The series connects career credibility to the ability to remain responsive and composed, even when private time and physical limits are under pressure.

Tang Ying, a lawyer, and Xu Ziquan, an investment banker, are shown within professional routines that divide time into appointments, client obligations, and opportunities for networking. Their expertise is valuable, but the narrative also shows how its value is tied to constant availability and the management of emotion.

The point is not that professional work eliminates agency. Rather, the dramas make agency conditional: characters must continually convert time, affect, appearance, and expertise into evidence of their employability. This conditionality gives the body a calculative role in the narrative.

Self-presentation also connects the body to consumption. Clothing, footwear, accessories, and grooming become visible cues through which characters anticipate how they will be read in professional and social settings.

In Nothing But Thirty and Fake It Till You Make It, designer handbags, shoes, and personal styling exceed practical function and become signs of class position and cultural distinction. Gu Jia’s Hermès handbag, for example, is presented as a condition for entering the wives’ circle and extending her business network. Consumption is therefore learned as a rule for judging both self and others.

Intimate relationships in Nothing But Thirty provide a third instance. Liang Zhengxian approaches romantic relations through appearance, wealth, and social status, while Wang Manni initially treats her cultivated image as a resource that may be converted into mobility. The narrative renders intimacy vulnerable to the same comparative logic found in consumption and work.

Objects and settings supply external standards; in work and intimate relations, the body converts them into everyday pressure. Reification here is not simply a matter of appearance being watched. Characters begin to treat their time, emotions, and manner of presentation as resources that require continuous maintenance. The body is therefore where the expectations established by objects and space are most readily internalized, and where a possible change in the relevant standards can first be observed.

### 4.4 Dereifying practice and the reconstruction of subjectivity

Meet Yourself is a contrastive case for dereifying practice. Its opening episodes locate Xu Hongdou in a performance-driven Beijing workplace; its later episodes move her to a rural community and reorganize her relation to time, care, and work. That movement allows the drama to imagine a partial reconstruction of subjectivity.

Care, community, and slower work are given material form through shared tasks, local cooperation, and renewed attention to friendship, family, and bodily limits. These practices do not cancel the pressures identified in the urban cases, but they make alternative relations narratively visible.

At the beginning of Meet Yourself, Xu Hongdou works as the front-office manager of a five-star Beijing hotel. Long hours, constant availability, and professional composure organize her daily life. Physical exhaustion, a relationship strained by lack of time, and a missed family occasion establish the personal cost of treating work pressure as the ordinary price of success.

The death of Xu Hongdou’s close friend Chen Nanxing interrupts that routine and produces a narrative break. Her decision to leave Beijing for Yunnan does not solve the social conditions described above; it changes the setting in which the character evaluates what she values and how she wants to live.

In Yunmiao Village, Xu Hongdou participates in the everyday work of a local guesthouse and in community activities. The narrative places shared work, reciprocal assistance, and unhurried conversation beside the earlier rhythm of performance and availability. Work is not abandoned, but its meaning is no longer limited to career advancement.

This does not mean that mutual assistance can resolve the problems of work, class, or consumption. Compared with the earlier dramas, the change lies in the fact that recognition no longer depends, at least temporarily, on acquiring the right object, entering a particular setting, or maintaining endless availability. Attention returns to fatigue, grief, friendship, and family life. Care takes on dereifying significance only when this shift changes how people are recognized and treated: shared work and mutual assistance allow concrete experience to enter relational judgement instead of being excluded by efficiency, status, exchangeability, or utility. The change remains at the level of everyday practice and narrative; it cannot be taken as evidence that labor relations or class structures have changed.

Here, people-centered narration does not simply praise a warm ending. It names a narrative choice: characters are no longer treated only as occupational or consumer subjects awaiting evaluation; their experience and relations again become part of the story. Meet Yourself makes such a choice imaginable, but it does not prove that reification has been overcome.

## 5 Discussion

The findings answer the research questions at connected levels. First, the selected dramas make commodity logic visible by translating social value into objects, addresses, bodily appearance, and professional performance. Second, routine consumption, spatial distinction, labor, and intimate evaluation reproduce that logic. Third, Meet Yourself imagines an interruption through reflective and relational practices rather than through a simple rejection of work or material needs. Read with Feenberg’s account of social practice (Feenberg, 2011, 2020), the corpus shows how television can make apparently fixed rules available for reflection within everyday experience.

Placing the three categories within a single relation makes it possible to see how an abstract judgement enters everyday life. A commodity sign first supplies a quick measure of value; space turns that measure into a condition of access and a rule of interaction; the body carries the rule into time, appearance, feeling, and intimacy. The three categories do not repeat the same point in different words. They perform distinct social work at different points in the formation of reified relations, and the process cannot be adequately explained if any one of them is removed.

The critical value of a warm, people-centered ending should nevertheless be qualified. Zheng (2024) notes that warm-realist dramas may draw attention to social problems while relocating their solutions to personal adjustment. The return to community, care, and meaningful work in Meet Yourself should thus be read as a cultural imagination of dereification, not proof that the institutions producing work pressure, class inequality, or consumer anxiety have changed. The argument is strongest when it retains the friction between structural constraint and individual agency.

The same qualification applies to care. Whether care has dereifying force depends on whether it changes the way a person is recognized: can fatigue, grief, dependence, and shared responsibility regain importance alongside speed, status, and utility? Reconstructing relations is, in this sense, a local revision of evaluative standards within everyday practice, not evidence that the larger system has escaped reification.

The study has clear limitations. Its small purposive corpus supports theoretical comparison rather than statistical generalization; its close readings cannot establish how viewers interpret or act on these representations; and it does not examine the production conditions that shape people-centered narration. Because the procedure is qualitative and interpretive, it does not make claims of coding reliability or frequency. Future work could combine comparative textual analysis with episode-level scene logs, audience interviews, production research, or platform data.

## 6 Conclusion

This article used qualitative close reading to examine human-object relations in three Chinese urban television dramas and one contrastive transition case. In the urban corpus, commodities, places, and bodies become media through which professional value, social status, and interpersonal worth are calculated and displayed. Reification is thereby rendered as a set of ordinary practices rather than as an abstract philosophical condition.

The article’s main theoretical contribution is to connect this representational analysis to Feenberg’s practice-oriented reading of Lukács. The cases show that dereification is narratively imagined when characters question the rules organizing efficiency, consumption, and exchange, and when they rebuild relations around care, shared activity, and meaningful work.

The relation among the three categories also sets limits on how the contrastive case should be read. Meet Yourself depicts a local change in how characters meet and evaluate one another: the pressure of commodity signs recedes for a time, the rules of interaction within space loosen, and the body no longer has to prove its availability without interruption. This does not mean that commodity signs, gatekeeping spaces, or pressure at work have ceased to operate.

### 5.1 Primary audiovisual sources

Fake It Till You Make It [Television series]. (2023). China.

Nothing But Thirty [Television series]. (2020). China.

Women in Beijing [Television series]. (2018). China.

Meet Yourself [Television series]. (2023). China.
